# Genetic background modifies phenotypic severity and longevity in a mouse model of Niemann-Pick disease type C1

**DOI:** 10.1242/dmm.042614

**Published:** 2020-03-13

**Authors:** Jorge L. Rodriguez-Gil, Dawn E. Watkins-Chow, Laura L. Baxter, Gene Elliot, Ursula L. Harper, Stephen M. Wincovitch, Julia C. Wedel, Arturo A. Incao, Mylene Huebecker, Frederick J. Boehm, William S. Garver, Forbes D. Porter, Karl W. Broman, Frances M. Platt, William J. Pavan

**Affiliations:** 1 Genomics, Development and Disease Section, Genetic Disease Research Branch, National Human Genome Research Institute, National Institutes of Health, Bethesda, MD 20892, USA; 2 Department of Pharmacology, University of Oxford, Oxford OX1 3QT, UK; 3 Medical Scientist Training Program, University of Wisconsin-Madison School of Medicine and Public Health, Madison, WI 53726, USA; 4 Embryonic Stem Cell and Transgenic Mouse Core, National Human Genome Research Institute, National Institutes of Health, Bethesda, MD 20892, USA; 5 Genomics Core, National Human Genome Research Institute, National Institutes of Health, Bethesda, MD 20892, USA; 6 Cytogenetics and Microscopy Core, National Human Genome Research Institute, National Institutes of Health, Bethesda, MD 20892, USA; 7 Department of Statistics, University of Wisconsin-Madison, Madison, WI 53706, USA; 8 Department of Chemistry and Chemical Biology, University of New Mexico, Albuquerque, NM 87131, USA; 9 Program in Developmental Endocrinology and Genetics, Eunice Kennedy Shriver National Institute of Child Health and Human Development, National Institutes of Health, Bethesda, MD 20892, USA; 10 Department of Biostatistics and Medical Informatics, University of Wisconsin-Madison, Madison, WI 53726, USA

**Keywords:** Niemann-Pick disease type C1, NPC1, Mouse models, Genetic modifiers

## Abstract

Niemann-Pick disease type C1 (NPC1) is a rare, fatal neurodegenerative disorder characterized by lysosomal accumulation of unesterified cholesterol and glycosphingolipids. These subcellular pathologies lead to phenotypes of hepatosplenomegaly, neurological degeneration and premature death. NPC1 is extremely heterogeneous in the timing of clinical presentation and is associated with a wide spectrum of causative *NPC1* mutations. To study the genetic architecture of NPC1, we have generated a new NPC1 mouse model, *Npc1^em1Pav^*. *Npc1^em1Pav/em1Pav^* mutants showed notably reduced NPC1 protein compared to controls and displayed the pathological and biochemical hallmarks of NPC1. Interestingly, *Npc1^em1Pav/em1Pav^* mutants on a C57BL/6J genetic background showed more severe visceral pathology and a significantly shorter lifespan compared to *Npc1^em1Pav/em1Pav^* mutants on a BALB/cJ background, suggesting that strain-specific modifiers contribute to disease severity and survival. QTL analysis for lifespan of 202 backcross N2 mutants on a mixed C57BL/6J and BALB/cJ background detected significant linkage to markers on chromosomes 1 and 7. The discovery of these modifier regions demonstrates that mouse models are powerful tools for analyzing the genetics underlying rare human diseases, which can be used to improve understanding of the variability in NPC1 phenotypes and advance options for patient diagnosis and therapy.

This article has an associated First Person interview with the first author of the paper.

## Abstract (precis)

**Summary:** This study identifies genomic regions in a new *Npc1* mutant mouse model containing potential modifier variants associated with changes in phenotypic severity and lifespan.

## INTRODUCTION

Niemann-Pick disease type C (NPC) is a rare, fatal neurodegenerative disorder that exhibits intracellular accumulation of unesterified cholesterol in late endosomes/lysosomes and marked accumulation of glycosphingolipids in neuronal tissue. NPC patients exhibit hepatosplenomegaly and neurodegeneration that invariably leads to premature death, yet the timing of these clinical presentations is variable, spanning prenatal stages to adulthood. Currently there are no Food and Drug Administration-approved therapies that effectively increase patient lifespan or slow NPC disease progression, although the substrate reduction therapy drug N-butyldeoxynojirimycin (miglustat, Zavesca) is approved in most non-North American countries worldwide and has shown potential for stabilizing neurological phenotypes and reducing glycosphingolipid levels (Fecarotta et al., 2015; Patterson et al., 2015; Stein et al., 2012). The incidence rate for NPC varies from 1 in 120,000 to 1 in 150,000 live births (Vanier and Millat, 2003). NPC has an autosomal recessive inheritance pattern, with most causative mutations (95%) located in the *NPC1* gene (NPC1 disease, OMIM: 257220), and the remainder (5%) located in the *NPC2* gene (NPC2 disease, OMIM: 607625) (Loftus et al., 1997; Naureckiene et al., 2000; Vanier and Millat, 2003). More than 500 disease-causing genetic variants have been identified in various protein regions for NPC1 and NPC2 (www.hgmd.org, accessed September 19th, 2019), of which I1061T, P1007A and G992W are the most frequent *NPC1* alleles (Millat et al., 1999; Vanier, 2010; Vanier and Millat, 2003). Although some mutations are frequently seen with specific phenotypic presentations, and some genotype-phenotype correlations are suggested based on patient analysis and cell culture studies, many exceptions exist (Benussi et al., 2015; Imrie et al., 2015; Millat et al., 1999, 2005, 2001; Shammas et al., 2019; Vanier, 2010; Vanier and Millat, 2003; Vanier et al., 1991; Walterfang et al., 2009). The context of a small patient population paired with the complexity of *NPC1* mutations makes analysis of underlying NPC1 genotype-phenotype correlations difficult. Furthermore, the phenotypic variation in NPC1 patients suggests genetic modifiers may be present in human NPC1 patients that contribute to variable clinical presentation. Different genetic backgrounds can also result in changes in phenotypic severity in *Npc1* mouse models. For example, differences in severity (Miyawaki et al., 1986), onset (Zhang and Erickson, 2000), survival (Liu et al., 2008; Marshall et al., 2018; Parra et al., 2011; Praggastis et al., 2015) and treatment response (Calderón and Klein, 2018) have all been reported, thus strongly suggesting that mouse models also carry genetic variants capable of modifying the NPC1 phenotype.

Owing to the rarity of NPC1 and its varied clinical presentations, the study of animal models is important for understanding the disease and developing effective therapies. Understanding the genetic interactions of *NPC1* with other genes and pathways that underlie the inherent phenotypic complexities of NPC1 disease will require the production of multiple different mouse models on a variety of genetic backgrounds. To date, there are eight publications describing spontaneous or targeted alleles affecting mouse *Npc1*. These include five alleles with a severe phenotype – the well-characterized *Npc1^m1N^* allele (widely known as *Npc1^nih^*), *Npc1^spm^*, *Npc1^pf^* (also known as *Npc1^tm1Mbjg^*), *Npc1^imagine^* and *Npc1^pioneer^* (Gómez-Grau et al., 2017; Loftus et al., 1997; Maue et al., 2012; Miyawaki et al., 1986; Xie et al., 2011). Two other mouse alleles, *Npc1^nmf164^* and *Npc1^I1061T^* (which recapitulates the most common mutation found in NPC1 patients), exhibit less severe phenotypes as a result of hypomorphic mutant alleles (Maue et al., 2012; Praggastis et al., 2015). In addition, the *Npc1^tm1.1Dso^* allele (also known as *Npc1^flox^*) allows tissue-specific deletion of *Npc1* (Elrick et al., 2010).

In this study, we used CRISPR/Cas9 to generate a novel *Npc1* mouse mutant, *Npc1^em1Pav^* (hereafter *Npc1^em^*), which harbors an in-frame deletion allele in the cysteine-rich domain of NPC1. These mice were generated and maintained on a C57BL/6J background, and *Npc1^em/em^* homozygotes recapitulated many characteristic phenotypes of NPC1 disease, including lipid storage abnormalities, visceral pathology and neurodegeneration resulting in a reduced lifespan. To identify genetic modifiers in NPC1, we evaluated the influence of genetic background on disease severity. Speed congenic techniques were used to establish intercross *Npc1^em/em^* mutant mice on a BALB/cJ background, then these mutants were analyzed at the N4 and N6 generations, when homozygosity for BALB/cJ had been attained at ≥92% of genotyped markers. These mutant mice showed a significantly increased lifespan and less severe visceral pathology when compared to the original C57BL/6J background. Analysis of N2 mice generated from a backcross using C57BL/6J and BALB/cJ found that *Npc1^em/em^* mutants also had an increased lifespan with greater variance, suggesting that strain-specific modifiers influenced disease severity. Genome-wide linkage analysis of 202 N2 mutants from this backcross detected significant linkage to regions on chromosome 1 and chromosome 7. These regions will provide candidate genes for future study as modifiers that may contribute to the highly variable phenotypes observed in NPC1 patients, thus advancing efforts to improve NPC1 patient diagnosis and therapy.

## RESULTS

### Generation of *Npc1^em^* mice

**Fig. 1.**
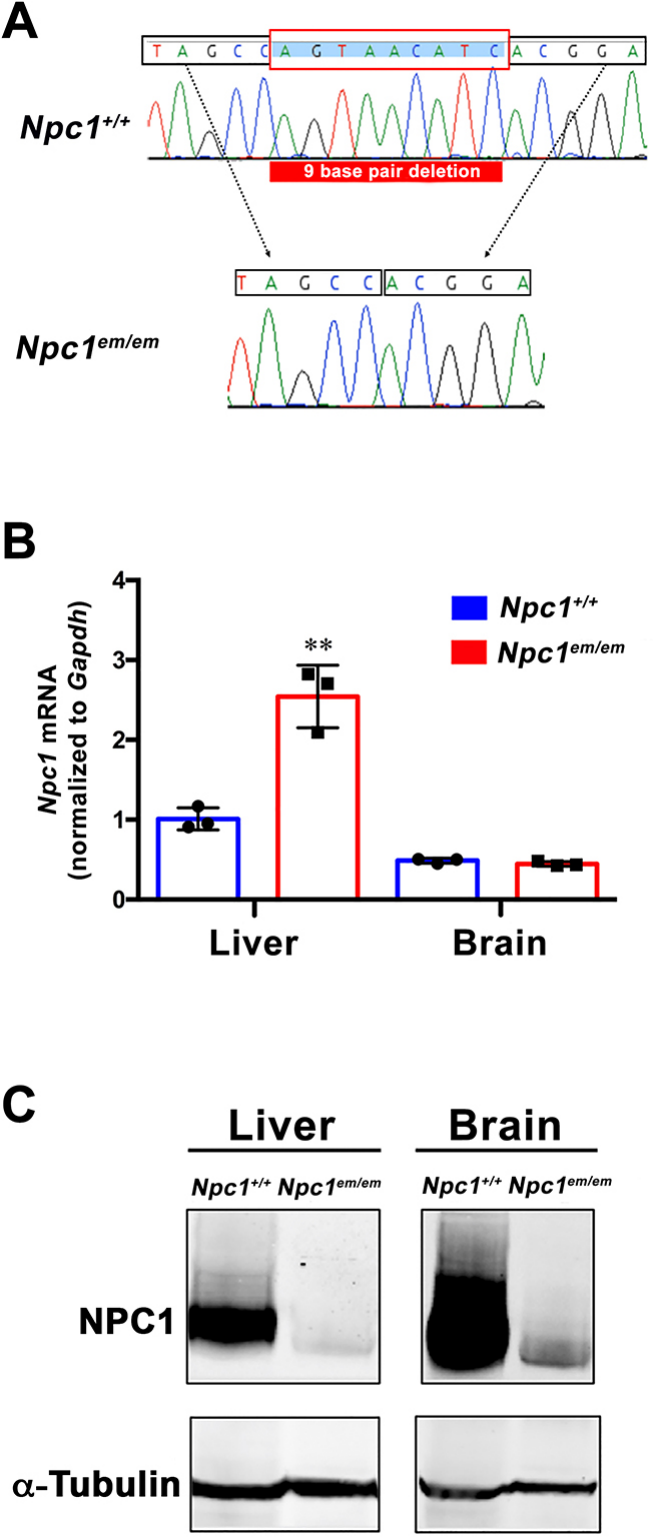
**Generation of the *Npc1^em^* allele and analysis of mRNA and protein levels in *Npc1^em/em^* mutant mice.** (A) Sanger sequencing results from *Npc1^em/em^* mice. The bottom chromatogram shows the *Npc1^em/em^* mutant genomic DNA sequence (missing the nine base-pair deletion, AGTAACATC) compared to the control *Npc1^+/+^* sequence on top. (B) RT-qPCR analysis using Taqman assays of *Npc1* mRNA normalized to *Gapdh*. Liver and brain tissues were collected from both *Npc1^em/em^* mutants (*n*=3) and control *Npc1^+/+^* littermates (*n*=3) at P68. Liver showed significantly higher levels of mRNA in the *Npc1^em/em^* mutants compared to controls (*P*=0.0031). *Npc1* mRNA levels in the brain were unchanged between the two groups (*P*=0.1749). **P≤0.01, unpaired Student's *t*-test. (C) Western blot analysis shows greatly reduced NPC1 protein levels in *Npc1^em/em^* mutants. Liver and brain tissues were harvested at 8 weeks from *Npc1^em/em^* mutants and control littermates (*Npc1^+/+^*).

The *Npc1^em^* allele was identified by screening founders from CRISPR/Cas9 injections of a single guide RNA targeted within the cysteine-rich loop domain of exon 21 of *Npc1*. Analysis of the *Npc1^em^*-associated mutation by Sanger sequencing revealed a nine-base pair in-frame deletion (Fig. 1A) that is predicted to result in deletion of three amino acids, Ser1062, Asn1063 and Ile1064 (S1062-I1064del). These amino acids reside in a highly conserved portion of the cysteine-rich domain of NPC1, in which more than one third of the identified human mutations are located (www.hgmd.org; Vanier and Millat, 2003). The *Npc1^em^* line was founded and maintained on a C57BL/6J inbred strain background. *Npc1^em^* was confirmed allelic to *Npc1* by a failure of complementation for the reduced lifespan phenotype in a complementation cross between *Npc1^em/+^* and *Npc1^nih/+^* heterozygous mice (Fig. S1).

Normalized levels of *Npc1* mRNA in the liver and brain of *Npc1^em/em^* and *Npc1^+/+^* controls were measured using qRT-PCR at ∼9 weeks of age. Interestingly, liver *Npc1* mRNA levels in *Npc1^em/em^* mutants were significantly increased compared to controls, but no difference in brain mRNA levels were observed (Fig. 1B). In contrast, protein analysis by western blot revealed a notably reduced amount of NPC1 in *Npc1^em/em^* homozygotes compared to control littermates that was evident in both brain and liver tissues (Fig. 1C). These results suggest the *Npc1^em^* allele acts as a severe hypomorph rather than a null, as *Npc1^em/em^* mutants still retained low levels of NPC1 protein.

### *Npc1^em/em^* mutants display lipid profiles, lysosomal storage pathology and neurodegenerative patterns consistent with NPC1 disease

**Fig. 2.**
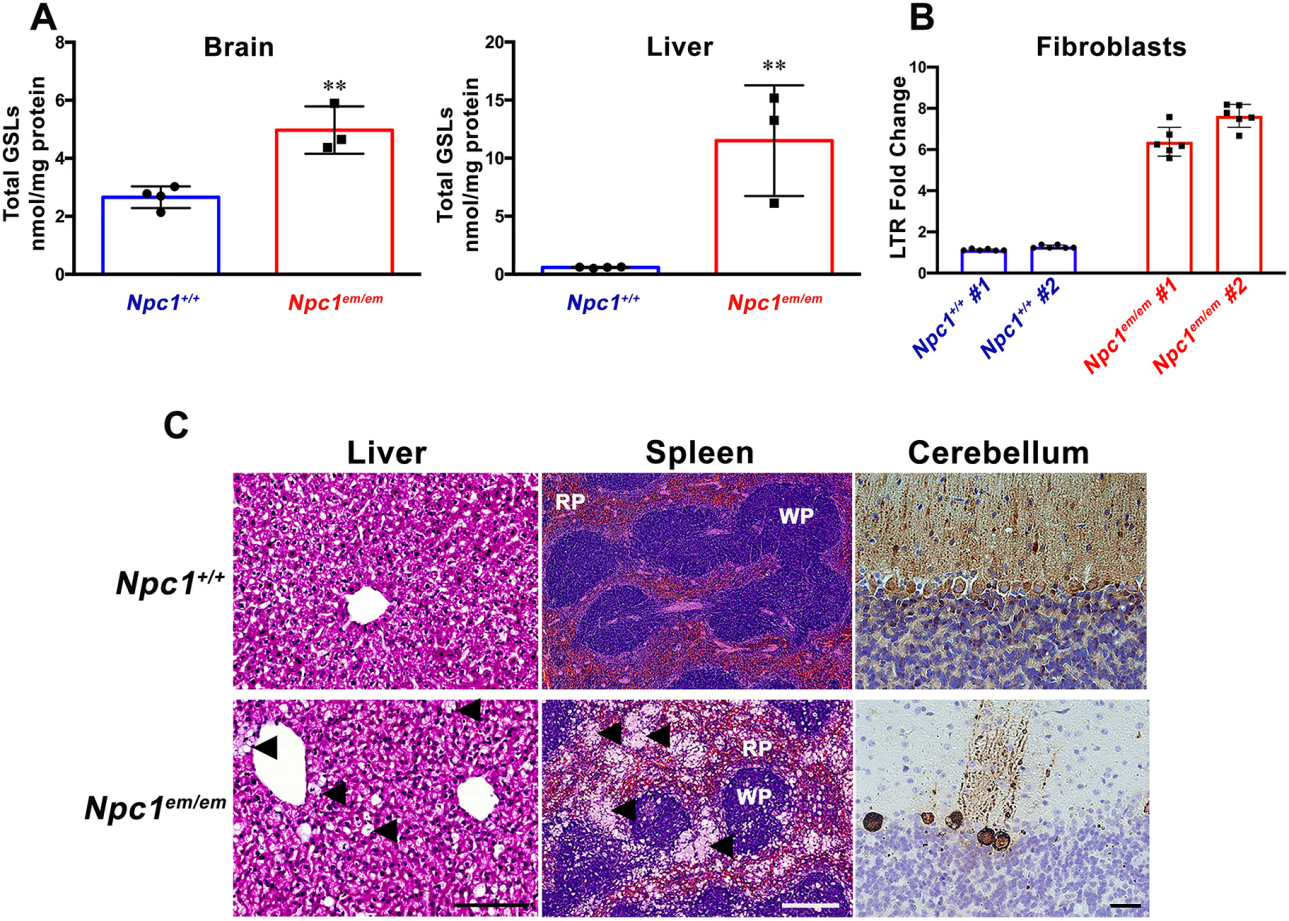
**Abnormal lysosomal function leads to lipid accumulation and storage pathology in *Npc1^em/em^ mice*.** (A) Brain and liver were collected from *Npc1^+/+^* controls (*n*=4) and *Npc1^em/em^* mutants (*n*=3) between P63 and P68. *Npc1^em/em^* mutants showed significantly elevated levels of total GSLs compared to controls in both the brain (*P*=0.0037) and liver (*P*=0.0051). All animals were on a C57BL/6J genetic background. Total GSLs (LacCer+GA2+Gb3+GM3+GM3Gc+Gb4+ GM2Gc levels) were measured in liver homogenates. Total GSLs (LacCer+GA2+Gb3+GM3+GM2+GA1+GM1a+GD1a+GD1b+GT1b+GQ1b) were measured in whole-brain homogenates. **P≤0.01, unpaired Student's *t*-test. (B) FACS results from primary fibroblasts that were derived from two different *Npc1^em/em^* mutants and two *Npc1^+/+^* controls (each labeled #1 and #2) were stained with LysoTracker (LTR). Data are mean±s.d. for six technical replicates for each primary fibroblast cell line. These results showed a significantly higher fold change in LTR staining in *Npc1^em/em^*-derived fibroblasts compared to controls (*P*=0.0117; nested Student's *t*-test). (C) Histological analysis of liver (left panels), spleen (center panels) and cerebellum (right panels) of *Npc1^+/+^* controls (top row) and *Npc1^em/em^* mutants (bottom row). In *Npc1^em/em^* liver, H&E staining showed numerous foam cells (indicated by arrowheads). In *Npc1^em/em^* spleen, H&E staining showed a foamy appearance due to lipid-laden macrophages (indicated by arrowheads) that resulted in architectural disruption of both the white pulp (WP) and red pulp (RP). In the cerebellum, calbindin staining (brown) revealed extensive loss of Purkinje neurons in *Npc1^em/em^* mutants compared to control littermates; Nissl (blue) staining of cerebellum. Scale bars: 100 µm for liver; 500 µm for spleen; 25 µm for cerebellum.

We performed a variety of biochemical and histological analyses to confirm that *Npc1^em/em^* mutants exhibited previously described phenotypes associated with NPC1 disease. For example, the accumulation of glycosphingolipids (GSLs) within visceral and neuronal tissue has been associated with NPC1 (Lloyd-Evans et al., 2010; te Vruchte et al., 2004; Zervas et al., 2001). Similarly, GSL analysis of brain and liver from *Npc1^em/em^* mutants showed a significant accumulation of total GSL (Fig. 2A) as well as individual GSL-related series (Fig. S2) in both tissues compared to controls. We have previously shown that higher levels of the well-known lysosomal marker LysoTracker are associated with NPC1 deficiency in fibroblasts and B cells of NPC1 patients (Rodriguez-Gil et al., 2013; te Vruchte et al., 2014). Similarly, primary fibroblasts derived from *Npc1^em/em^* mutants showed significantly increased LysoTracker staining compared to controls (Fig. 2B**)**. Histopathology of the liver, spleen and cerebellum of *Npc1^em/em^* mice showed similar findings to other *Npc1* mouse mutants (Gómez-Grau et al., 2017; Loftus et al., 1997; Maue et al., 2012; Miyawaki et al., 1986; Praggastis et al., 2015; Xie et al., 2011), including the presence of foam cells in both the liver and spleen as well as Purkinje neuron loss in the cerebellum (Fig. 2C).

### *Npc1^em/em^* mutants display weight loss, progressive motor impairment and reduced lifespan

**Fig. 3.**
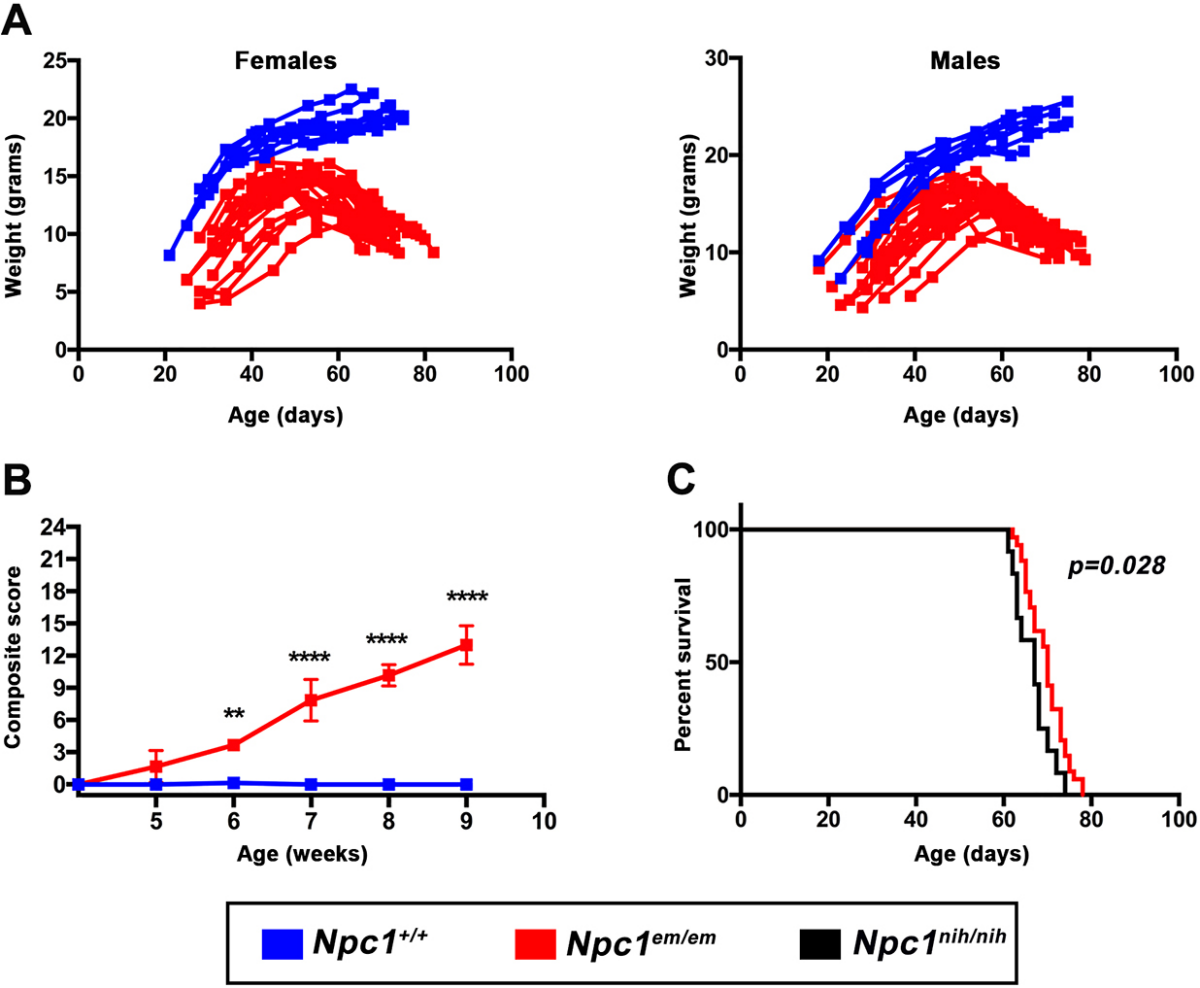
**Progressive weight loss and neurodegenerative phenotypes are accompanied by reduced lifespan in *Npc1^em/em^* mice.** (A) Average weight loss onset occurred at 59.8 days, ranging from 58 to 66 days for females (left) and 60.7 days, ranging from 55 to 66 days for males (right). *Npc1^+/+^* controls (blue), *n*=8 females, *n*=9 males; *Npc1^em/em^* mutants (red), *n*=19 females, *n*=21 males. (B) Progressive neurodegeneration and motor impairment in *Npc1^em/em^* mutants (*Npc1^em/em^* mutants, *n*=6; *Npc1^+/+^* controls, *n*=7). A combination of six different neurodegenerative phenotypes and behavioral tests were scored weekly starting at weaning (P28) and combined into a composite score (see Materials and Methods). A higher score means greater severity; *Npc1^em/em^* mutant scores were significantly different than *Npc1^+/+^* controls. **P≤0.01, ****P≤0.0001 (two-way ANOVA with repeated measures and Bonferroni's correction; *P*<0.0001 for time, genotype and interaction). (C) Survival analysis of *Npc1^em^* and *Npc1^nih^* alleles on a C57BL/6J background. Survival of *Npc1^em/em^* mice was greatly reduced compared to *Npc1^+/+^*, with a median survival of 70 days (*n*=34). Furthermore, the survival of *Npc1^em/em^* mice was slightly increased in comparison to mice homozygous for the *Npc1^nih^* null allele [67 days, *n*=12; *P*=0.028, log-rank (Mantel-Cox) test]. Gender composition for both groups was: *Npc1^nih/nih^* (females, *n*=4; males, *n*=8), *Npc1^em/em^* (females, *n*=14; males, *n*=20).

*Npc1^em/em^* mutants showed progressive weight loss in both males and females starting at ∼59 days of age (Fig. 3A). One of the phenotypic hallmarks of *Npc1* mouse models is progressive motor impairment, which manifests as ataxia and changes in gait. Starting at 4 weeks of age, *Npc1^em/em^* mutants and age-matched *Npc1^+/+^* controls were given a weekly composite score that measured motor abnormalities, generated from the sum of scores given for the following six categories, as previously described (Alam et al., 2016; Guyenet et al., 2010): grooming, gait, kyphosis, ledge test, hindlimb clasp and tremors (Fig. 3B). As the disease progressed, the *Npc1^em/em^* composite score became significantly higher than that of controls. The earliest noticeable *Npc1^em/em^* phenotype was change in gait accompanied by kyphosis, whereas hindlimb clasping was observed at a later stage of the disease (week 9). *Npc1^em/em^* mice also showed a significantly reduced lifespan in comparison to *Npc1^+/+^* mice, with a mean survival of 70 days (Fig. 3C). *Npc1^em/em^* mice also showed a small increase in lifespan in comparison to that of *Npc1^nih/nih^* null mice (67 days, *P*=0.028) on the same C57BL/6J genetic background (Fig. 3C). These results confirm that *Npc1^em/em^* mice exhibited a reduced lifespan and other phenotypes that are associated with neurodegeneration and motor impairment that have been previously observed in other *Npc1* mouse mutants.

### Severity of visceral pathology and lifespan of *Npc1^em/em^* mutants is modified by genetic background

Previous studies have suggested that genetic background influences disease severity and lifespan in *Npc1* mutant mice (Miyawaki et al., 1986; Parra et al., 2011; Praggastis et al., 2015; Zhang and Erickson, 2000). To further test this hypothesis, marker-assisted selection/speed congenic techniques were used to rapidly establish a congenic strain of the *Npc1^em^* allele on a BALB/cJ genetic background. This experimental design allowed production of *Npc1^em/em^* mice that exhibited ≥92% BALB/cJ homozygosity for all tested genetic markers by the N4 generation (see Materials and Methods).

**Fig. 4.**
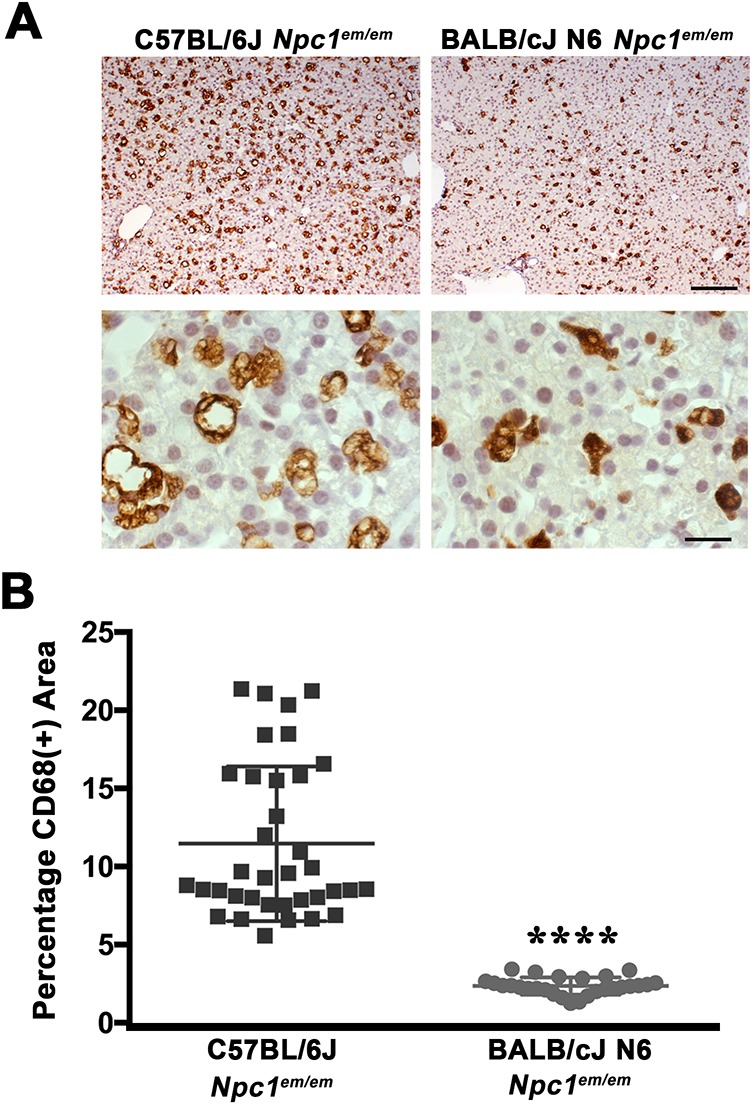
**Visceral pathology in *Npc1^em/em^* mutants varies with genetic background.** (A) Liver tissues from age-matched (P21) *Npc1^em/em^* mutants on a C57BL/6J genetic background (left) or a BALB/cJ N6 intercross genetic background (right). Tissues were stained with the macrophage marker CD68 (brown). *Npc1^em/em^* mutants on a C57BL/6J genetic background showed a greater accumulation of foam cells compared to the *Npc1^em/em^* mutants on a BALB/cJ N6 intercross genetic background. A higher magnification view (bottom row) shows that the foam cells appeared to be larger in size owing to lipid storage. (B) Quantification of CD68^+^ signal showed a significant difference between a C57BL/6J genetic background and a BALB/cJ N6 intercross genetic background. ****P<0.0001, unpaired Student's *t*-test. Each dot represents the average of nine independent fields within an ROI, and nine ROIs were analyzed for each animal. C57BL/6J, *n*=4; BALB/cJ, *n*=3. Scale bars: 200 µm, top row; 10 µm, bottom row.

Liver foam cells, as measured by staining for the macrophage marker CD68 (CD68^+^), were examined in age-matched *Npc1^em/em^* mutants [postnatal day (P)21] on a C57BL/6J genetic background and compared with congenic BALB/cJ *Npc1^em/em^* mutants at the N6 intercross generation (BALB/cJ N6, Fig. 4A). *Npc1^em/em^* mutants on a C57BL/6J genetic background exhibited foam cell accumulation that was significantly greater than that of *Npc1^em/em^* mutants on the BALB/cJ N6 background (Fig. 4A). Quantification of the percentage area of CD68^+^ signal in multiple *Npc1^em/em^* mutants confirmed the presence of a more severe storage phenotype in *Npc1^em/em^* mutants with a C57BL/6J genetic background (Fig. 4B). These results indicate that underlying cellular pathologies, such as immunological responses due to lipid accumulation that are indicated by CD68^+^ cells, are affected by strain-specific changes.

**Fig. 5.**
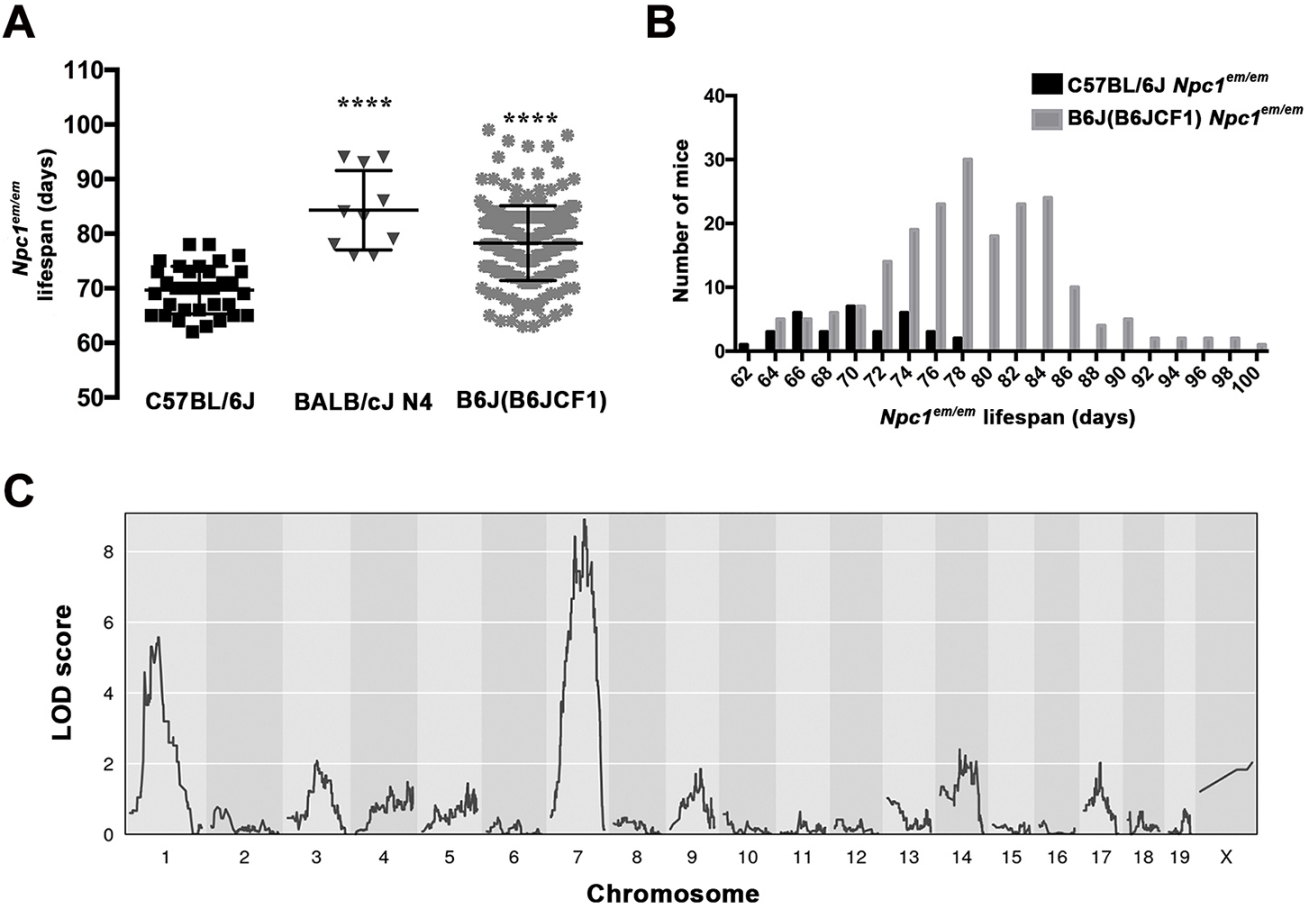
**Strain-specific lifespan differences in *Npc1^em/em^* mutants map to chromosomes 1 and 7.** (A) Lifespan was measured for *Npc1^em/em^* mutants on three different genetic backgrounds: C57BL/6J, BALB/cJ N4 intercross and N2 backcross [B6J(B6JCF1)]. Median survivals were: C57BL/6J, 70 days (*n*=34); BALB/cJ N4 intercross, 83.5 days (*n*=10); N2 [B6J(B6JCF1)] backcross, 78 days (*n*=202). The N2 backcross *Npc1^em/em^* mutants showed a wide range in lifespan, spanning the extremes of both the *Npc1^em/em^* C57BL/6J and *Npc1^em/em^* BALB/cJ N4 intercross mutants. Mean±s.d. values were: *Npc1^em/em^* C57BL/6J, 69.7±4.4 days; *Npc1^em/em^* BALB/cJ N4 intercross mutants, 84.3±7.3 days; *Npc1^em/em^* N2 backcross, 78.3±6.8 days. ****P<0.0001, one-way ANOVA. Each dot represents an individual animal. (B) Frequency distribution of lifespan in *Npc1^em/em^* mutants on a C57BL/6J background (*n*=34, black) shows distribution for the population is centered at 70 days. In contrast, frequency distribution of N2 [B6J(B6JCF1)] *Npc1^em/em^* mutants (*n*=202, gray) shows a greater range with a mean at 78 days, which is located at the high end of the C57BL/6J population. (C) Genetic linkage results between lifespan of *Npc1^em/em^* mutants and strain-specific markers for C57BL/6J and BALB/cJ. Genome scan results from the B6J(B6JCF1) N2 backcross generation by individual chromosome locations identified areas with significant LOD scores on chromosome 1 (LOD=5.57) and chromosome 7 (LOD=8.91). The marker with the highest score (peak) at chromosome 1 was UNC99871 at 40.76 cM, and at chromosome 7 was UNC13374196 at 51.5 cM. Analysis was performed with a 5% significant threshold of LOD>3.09.

To determine whether the severity of other phenotypes associated with NPC1 such as lifespan were also affected by changes in genetic background, the lifespan of *Npc1^em/em^* mutants on a C57BL/6J background was compared to the lifespan of congenic BALB/cJ *Npc1^em/em^* mutants at the N4 generation (BALB/cJ N4). The BALB/cJ N4 *Npc1^em/em^* mutants showed a significantly longer lifespan than *Npc1^em/em^* mutants on a C57BL/6J background (*P*<0.0001, Fig. 5A). Taken together, these results support the hypothesis that strain-specific variants between C57BL/6J and BALB/cJ play a role in NPC1 survival and disease severity.

### QTLs on chromosomes 1 and 7 are associated with survival in *Npc1^em/em^* mutants

Given the strain-specific differences in survival of *Npc1^em/em^* mice, we next measured the lifespan of mice with a mixed genetic background for C57BL/6J and BALB/cJ, to facilitate mapping genetic modifiers of lifespan. To do this, a traditional backcross of BALB/cJ males to C57BL/6J *Npc1^em/+^* females was performed, and subsequently the lifespan of *Npc1^em/em^* mutants from the N2 backcross generation [B6J(B6JCF1)] was measured. The lifespan of *Npc1^em/em^* N2 backcross mutants (*n*=202) was significantly longer than that of C56BL/6J *Npc1^em/em^* mice (*P*<0.0001) and also showed a wide range, overlapping with the lifespan of both the C56BL/6J *Npc1^em/em^* mice and BALB/cJ N4 intercross *Npc1^em/em^* mice (Fig. 5A). The frequency distribution for lifespan for the C57BL/6J *Npc1^em/em^* population ranged from 62 to 78 days (Fig. 5B); in contrast, the distribution for N2 backcross mice showed a greater range, from 63 to 99 days, with a mean located at the high end of the C57BL/6J population (78 days, Fig. 5B). This broad distribution of lifespan in N2 mice suggests the presence of multiple genetic modifiers affecting lifespan in *Npc1^em/em^* mutants.

To identify potential modifiers of *Npc1^em/em^* mutant phenotypes, we performed quantitative trait locus (QTL) linkage analysis using lifespan as the phenotype for the 202 *Npc1^em/em^* mice in the B6J(B6JCF1) N2 backcross generation. Genotype results were analyzed using 28,873 genome-wide single-nucleotide polymorphisms (SNPs) that were informative between C57BL/6J and BALB/cJ. Linkage results (Fig. 5C) revealed markers associated with significant LOD scores on chromosome 1 (Marker ID: UNC99871, 40.76 cM; LOD=5.57) and chromosome 7 (Marker ID: UNC13374196, 51.5 cM; LOD=8.91). When controlling individually for the effects of the chromosome 1 and chromosome 7 QTLs, we found no evidence of a secondary QTL in the same area nor a change in the effect of the other QTL (Fig. S3A**)**. To examine a possible epistatic interaction between the two QTL regions, the N2 backcross samples were separated into four possible genotypes. This showed that the effect of the QTL region on chromosome 1 does not depend on the genotype of chromosome 7 and vice versa, suggesting additivity (Fig. S3B). This analysis also showed that homozygosity for C57BL/6J on chromosome 7 resulted in a shorter lifespan compared with mice heterozygous for BALB/cJ in the same region. In contrast, chromosome 1 showed the opposite effect, with C57BL/6J homozygosity in this region resulting in a longer lifespan. Thus, *Npc1^em/em^* mutants with both C57BL/6J homozygosity at chromosome 1 and heterozygosity for BALB/cJ and C57BL/6J on chromosome 7 showed the longest lifespan.

Boundaries were established for both QTLs using a region in which the LOD score is within 1.5 of the highest score (peak), as previously recommended (Manichaikul et al., 2006). These QTL intervals were flanked by the following markers: chromosome 1, UNC484527 (17.241 cM; 38,919,200 bp) to UNC112857 (45.334 cM; 92,082,500 bp) and chromosome 7, UNC12909197 (33.483 cM; 63,988,100 bp) to UNCHS020933 (55.703 cM; 111,771,000 bp). Thus, the two intervals spanned 53.2 Mbp for chromosome 1 and 47.8 Mbp for chromosome 7. The Mouse Genomes Project (Sanger; release Rel1303-GCRm38, https://www.sanger.ac.uk/sanger/Mouse_SnpViewer/rel-1303) was used to perform a SNP inquiry between C57BL/6J and BALB/cJ for these regions, filtering for variants predicted to impact coding sequences. This analysis identified multiple candidate genes and regions for future analyses (Table 1, Table S1). Of note, when we controlled for the effects of both the chromosome 1 and chromosome 7 QTL regions, one additional, lower-scoring QTL was identified on chromosome 17 (LOD>3, Fig. S3C**)**, suggesting that additional genomic regions may make minor contributions to the complex modifier effects on NPC1 phenotypes. In summary, these results suggest that strain-specific survival changes in *Npc1^em/em^* mutants are due to contributions of multiple modifier genes that impact lifespan in the context of NPC1 disease.

**Table 1.**
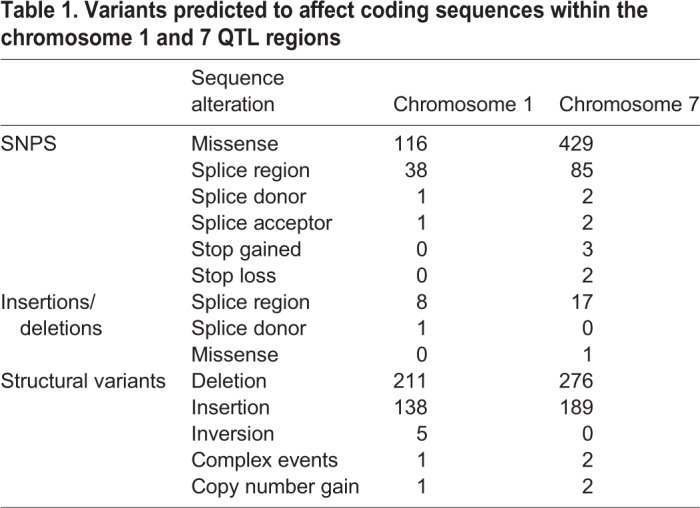
**Variants predicted to affect coding sequences within the chromosome 1 and 7 QTL regions**

## DISCUSSION

Animal models are important tools to use for evaluation of rare, fatal disorders such as NPC1, because the small size of the affected patient population is often insufficient to power conventional clinical trials (Miller et al., 2018). NPC1 also presents the additional complexities of extensive genetic and clinical heterogeneity, including phenotypic differences that are present even among patients with the same *NPC1* mutation (Vanier, 2010; Vanier et al., 1991, 1988; Walterfang et al., 2009). This heterogeneity will require multiple animal models carrying different alleles to reproduce and subsequently analyze these differences (Fog and Kirkegaard, 2019). Furthermore, although advances in sequencing technology have identified many genes responsible for Mendelian disorders (Bamshad et al., 2011), further analysis of the effects of these mutations – including *NPC1* mutations – has revealed that phenotypic variation still exists, even when accounting for their primary causative effect (Dipple and McCabe, 2000; Scriver and Waters, 1999; Vanier et al., 1988, 1991; Walterfang et al., 2009). This suggests that the term ‘monogenic disorder’ may be too simplistic to describe the phenotypic differences among affected individuals with the same disease-causing mutations, and not take into account the importance of genetic modifiers (Nadeau, 2001; Riordan and Nadeau, 2017). In this paper, we have provided data that address both the need for additional NPC1 animal models and the need to expand knowledge of genetic modifiers underlying the complexities associated with NPC1 phenotypes, by identifying two previously unidentified chromosomal regions that modify NPC1 phenotypic severity using a newly generated *Npc1* mouse model.

The *Npc1^em/em^* mutant is the first NPC1 mouse model generated using CRISPR/Cas9 targeting, and also the first *Npc1* in-frame deletion allele in mouse. This mutation is located in the cysteine-rich loop domain of the protein in which most NPC1 human mutations occur. Although the specific *Npc1^em^* mutation does not recapitulate a known human *NPC1* allele to date, we chose to pursue detailed analysis of *Npc1^em/em^* mice because they showed classical phenotypes associated with NPC1, thus implicating these amino acids as critical for NPC1 function.

Western blot analysis from both liver and brain indicated that *Npc1^em/em^* mutants expressed residual NPC1 protein. In addition, *Npc1^em/em^* mutants have a small increase in lifespan when compared to the *Npc1^nih^* null allele; future studies using bigger sample sizes will be needed to validate these observations. These results suggest that *Npc1^em^* may act as a severely hypomorphic rather than null allele. Interestingly, *Npc1* mRNA levels from *Npc1^em/em^* mutants did not correlate with diminished NPC1 protein levels, as mutant mRNA levels were equal to control levels in brain and were actually higher in the liver. Previous publications have also shown no correlation between the levels of mutant *Npc1* transcript and the amount of mutant protein in homozygous I1061T patient-derived fibroblasts and in the I1061T mouse model (*Npc1^tm1.1Dso^*; Gelsthorpe et al., 2008; Praggastis et al., 2015). These data suggest mutant *Npc1* may be regulated at the transcriptional level in a tissue-specific manner, possibly by a feedback mechanism in response to lower protein levels, or post-translationally, as has been indicated by a higher molecular weight of mutant NPC1 protein in the *Npc1^tm1.1Dso^* mouse model (Praggastis et al., 2015). These findings could be important for future targeted mRNA stability treatments such as antisense oligonucleotides (Rinaldi and Wood, 2018) or chaperone-mediated treatments (Kirkegaard et al., 2016; Penke et al., 2018).

Multiple analyses indicated that *Npc1^em/em^* mutant mice are able to recapitulate many aspects of NPC1 disease, thus making it an appropriate NPC1 model. GSL accumulation in visceral and neuronal tissue, which has been associated with NPC1 (Maue et al., 2012; te Vruchte et al., 2004), also occurred in the brain and liver of *Npc1^em/em^* mutants. In addition, elevated staining with the lysosomal marker LysoTracker is associated with NPC1 disease (Rodriguez-Gil et al., 2013; te Vruchte et al., 2014), and primary fibroblasts from *Npc1^em/em^* mutants also showed increased LysoTracker staining. To the best of our knowledge, this is the first publication showing LysoTracker staining [measured by fluorescence-activated cell sorting (FACS)] in *Npc1* mouse mutant fibroblasts derived from skin/ear. This constitutes a non-invasive assay to test lysosomal dysfunction in NPC1, which can be used for future studies to test different therapeutic approaches in a more rapid and quantifiable manner. Accumulation of lipids in the reticuloendothelial system in NPC1 disease leads to the presence of foam cells in peripheral tissues. These lipid-laden macrophages become enlarged and are easily distinguished in liver and spleen by hematoxylin and eosin (H&E) staining, and our pathology analyses showed this classical foamy appearance in both liver and spleen from *Npc1^em/em^* mutants. One of the main clinical presentations of NPC1 patients is cerebellar ataxia. Analysis of the cerebellum of *Npc1^em/em^* mutants showed loss of Purkinje neurons, which led to progressive motor impairment and neurological abnormalities in *Npc1^em/em^* mutants that were detected and quantified by behavioral tests.

As mentioned above, understanding the role of genetic modifiers will be key to elucidating the phenotypic complexity of NPC1 disease. Interestingly, we found that the genetic background of *Npc1^em/em^* mice had a significant effect on phenotypic severity that led to changes in NPC1-associated longevity. This corresponded with previous evidence showing strain-specific differences in survival and disease onset (Liu et al., 2008; Marshall et al., 2018; Miyawaki et al., 1986; Parra et al., 2011; Praggastis et al., 2015; Zhang and Erickson, 2000). Although these previous studies suggested that a genetic component contributed to these phenotypic variations, the identification of these modifier genes was still unknown. Thus, we designed experiments to locate genomic regions harboring NPC1 modifier variants by using the naturally occurring genetic differences found between mouse strains.

We generated and maintained the new *Npc1^em^* hypomorphic allele on a C57BL/6J genetic background to allow comparison of disease-related phenotypes with other strains, such as BALB/cJ. By using marker-assisted genotyping (speed congenics), *Npc1^em/em^* mutants with a high percentage of BALB/cJ homozygosity were quickly established by targeting male breeders carrying the highest percentage of the desired inbred strain, thus obtaining a congenic strain in only five generations (∼1.25 years) compared to a traditional backcross which can take up to ten generations (∼2.5 years). We showed that the *Npc1^em/em^* BALB/cJ congenic strain exhibited less severe liver pathology and a longer lifespan as compared to *Npc1^em/em^* C57BL/6J mice. Furthermore, measurement of lifespan from a genetically mixed N2 generation of *Npc1^em/em^* mutants [B6J(B6JCF1)] gave a broad distribution that indicated the presence of multiple modifier genes.

Our QTL analysis of the B6J(B6JCF1) N2 generation showed two significant QTLs affecting lifespan on chromosome 1 and chromosome 7. Further analysis was consistent with an additive effect on lifespan between these two regions rather than an epistatic interaction. Interestingly, these results showed that C57BL/6J homozygosity on chromosome 1 contributes to increased survival. Our analysis also suggested heterozygosity for BALB/cJ at the chromosome 7 QTL will result in increased lifespan. This phenomenon fits in part with previous reports showing a BALB/cJ genetic background increases lifespan in both *Npc1^nih^* and *Npc1^I1061T^* mice compared to C57BL/6J mutants (Parra et al., 2011; Praggastis et al., 2015). Furthermore, we also identified another potential QTL on chromosome 17 (LOD>3) when controlling for the effects of chromosome 1 and 7. These results underscore the complexity and multifactorial nature of the genetic architecture of NPC1 disease. In general, sample size is considered a primary limiting factor in QTL analysis, and additional QTL regions affecting lifespan could potentially be identified with a greater sample size. However, the number of *Npc1^em/em^* mutants from the N2 generation in this study (*n*=202) was large enough to reveal two QTLs with highly significant LOD scores, suggesting a sufficient sample size was used for analysis of this NPC1 phenotype.

To our knowledge, our study is the first to identify genomic regions in *Npc1* mutant mice containing potential modifier variants associated with changes in lifespan, thus showing an underlying genetic component that contributes to part of the phenotypic variation in survival in *Npc1* mutant mice. We also showed that these genetic modifiers can have independent effects as well as interact with each other in an additive manner. These results differ from a previously published QTL study, in which the age of onset of tremor was used as the phenotype in *Npc1* mutant mice from a mixed (BALB/cJ and DBA2/J) N2 generation, and linkage (LOD=2.24) was detected on chromosome 19 (Zhang and Erickson, 2000). The differences between the QTL regions in our study and the results from Zhang and colleagues could be due to the analysis of different phenotypes (age of onset versus lifespan) and/or the use of different genetic strains. Nevertheless, these previously published results suggested, as in our case, the presence of multiple genetic modifiers based on the distribution of the N2 generation.

Future work will focus on generating a list of candidate genes based on strain-specific variants between C57BL/6J and BALB/cJ. For example, the most deleterious strain-specific variants can be selected based on conserved sequences (Choi et al., 2012; Eilbeck et al., 2017) and then prioritized based on expression in known NPC1-affected organs such as liver, brain and spleen. Ultimately, the most promising candidates will be tested *in vivo* using CRISPR/Cas9 to generate mutant mouse strains for each candidate. These variants can be introduced separately in *Npc1^em/em^* mutant alleles and their phenotypic effect on survival and other disease phenotypes can be characterized. These results could also be combined with transcriptional analysis and whole exome sequencing datasets generated from NPC1 patients. The combination of multiple datasets from patient populations and animal models will allow prioritization of candidate genes that are relevant in NPC1 patients and thus have the potential to modulate the disease, working towards the urgent need to identify new treatments to alleviate NPC1 disease phenotypes. Importantly, as many of these pathogenic pathways are common to other lysosomal storage disorders (Platt et al., 2018), the identification of these genetic modifiers may also be applicable to other rare disorders.

## MATERIALS AND METHODS

### Generation of *Npc1^em1Pav^* mice

*Npc1* was targeted within the cysteine-rich loop domain of exon 21 on a C57BL/6J genetic background, using a 20 bp CRISPR/Cas9 target sequence (GCTAATAGCCAGTAACATCA) selected with publicly available software tools (Benchling.com). Potential off-target regions were analyzed based on the target sequence. No off-target sites were detected linked to *Npc1* on chromosome 18 (Table S2). Oligonucleotides with a linked T7 site and Cas9 scaffold sequence were synthesized, annealed and filled in to generate a double-stranded template for guide (g)RNA synthesis using the MEGAshortscript T7 Kit (Thermo Fisher Scientific). gRNA was subsequently purified using the MEGAclear Transcription Clean-Up Kit (Thermo Fisher Scientific) according to the manufacturer's instructions. RNA quality was assessed using a Bioanalyzer instrument (Agilent Genomics) and stored in aliquots at −80°C until used for injections. Pronuclear injection into *Mus musculus* C57BL/6J fertilized eggs was performed with standard procedures, as previously described (Watkins-Chow et al., 2017), and founders were screened using a combination of PCR and sequencing. A PCR product spanning the gRNA target site was generated using an *Npc1*-specific forward primer with an M13-tail (5′-tgtaaacgacggccagtTGAGAGCGAAGGATCTGCAGTC), and an *Npc1*-specific reverse primer containing a pig-tail (5′-gtgtcttGGGGCCACTTACTTCATGACCT). The PCR product was amplified with the addition of a 6-FAM- or HEX-labeled M13-forward oligonucleotide and run on an Applied Biosystems (ABI) 3130xl with ROX400 or ROX500 size standards to detect small indels at single base pair resolution by capillary electrophoresis. Standard agarose gels were also used to screen PCR products for larger indels. A male founder was identified with a small indel within the cysteine- rich loop domain and used to establish the *Npc1^em1Pav^* colony (abbreviated throughout the manuscript as *Npc1^em^*). The precise mutation was further characterized by Sanger sequencing (Eurofins Genomics) of PCR products generated with additional gene-specific primers (F: CTCTCCTGTGACTCTCTGGG and R: AGCTGTGCATCATGTTTGGT) and confirmed in multiple offspring of the original founder.

### Nomenclature

Standard nomenclature of the allele was registered as *Npc1^em1Pav^* following the Guidelines for Nomenclature of Genes, Genetic Markers, Alleles, and Mutations in Mouse and Rat approved by the International Committee of Standardized Genetic Nomenclature for Mice (Mouse Genome Informatics).

### Colony management and genotype identification

Colonies were maintained by following the standard protocol of the Institutional Animal Care and Use Committee from the National Human Genome Research Institute (NHGRI). There were between two and five adult mice per cage (regardless of their genotype). Backcrossing to C57BL/6J (Stock number: 0000664, Jackson Laboratories) was used to maintain the *Npc1^em^* colony and heterozygotes were mated to generate homozygote mice for analysis. DNA was extracted from pup tail biopsies at P10 and purified using a Gentra Puregene Mouse Tail Kit (Qiagen). To date, we have bred more than 10 generations of the *Npc1^em^* mouse colony from the founder mutant male. Genotyping was performed using the Custom Taqman Assay Design Tool (Thermo Fisher Scientific). Samples were amplified using the following primers and probes using a Universal 2× Taqman Master Mix (Thermo Fisher Scientific) and an ABI 7500 instrument for thermocycling and detection: FAM, TTACTGGCTGTTAGCCG-MGBNFQ; VIC, ATGTTACTGGCTATTAGCCG-MGBNFQ; F, GCGGTAGTCACTCCCCTTAG; R, CCATGAAGAAAGCTCGGCTA. Genotyping for the *Npc1^em^* allele can also be performed using gel electrophoresis with an expected amplification of 150 base pairs, using the following primers: Forward, CACCTGTAAGGGAATACGCGG; Reverse, GGCCACTTACTTCATGACCT. *Npc1^m1N^* mice (referred to as *Npc1^nih^* throughout the manuscript) were genotyped as previously described (Loftus et al., 1997).

### qRT-PCR (mRNA quantification)

Tissues were harvested and immediately homogenized in 1 ml Trizol (Thermo Fisher Scientific). We added 200 μl of chloroform, and the recovered supernatant was loaded onto a silica-membrane column for purification following the manufacturer's instructions (RNeasy Kit, Qiagen). cDNA was synthesized using 1μg of total RNA per reaction using a High-Capacity cDNA Reverse Transcription Kit (Thermo Fisher Scientific). Taqman gene expression assays (Thermo Fisher Scientific) were used to amplify *Npc1* (Assay ID: Mm00435300_m1) and *Gapdh* (Assay ID: Mm99999915_g1). A control cDNA was used in serial dilutions to generate a standard curve and calculate the relative expression level of each gene. Real-time PCR reactions were performed using a StepOne machine (ABI). Each sample was analyzed using three technical replicates in a 96 well plate. Technical replicates were averaged, and the expression level of *Npc1* was normalized to the housekeeping gene *Gapdh*.

### Western blot

Tissue lysates were prepared by incubating them with RIPA buffer [150 nM sodium chloride, 1% Triton X-100, 0.5% sodium deoxycholate, 0.1% sodium dodecyl sulfate (SDS), 50 mM Tris (pH 8.0)] for 2 h at 4°C with 10× protein inhibitor cocktail (Millipore Sigma). Samples were centrifuged for 20 min at 13,523 ***g*** on a table centrifuge (Eppendorf 5424, 24-place aerosol). Supernatant was separated and loaded with 5% β-mercaptoethanol (BME; Millipore Sigma) and 2× Tris-Glycine SDS sample buffer (Thermo Fisher Scientific) on a 4-12% Bis-Tris protein gel (Thermo Fisher Scientific). Protein was transferred onto a nitrocellulose membrane (Thermo Fisher Scientific) using an iBLOT dry transfer system (Thermo Fisher Scientific) with an 8 min transfer time. Primary antibodies for NPC1 (134113, Abcam) and α-tubulin (CP06, Millipore Sigma) were diluted 1:1000 and were incubated overnight at 4°C in blocking buffer (LI-COR Biosciences) with TBST (Tris-buffered saline with 0.1% Tween 20) (T9039, Millipore Sigma). Blots were washed three times in PBST before incubating for 1 h at room temperature with secondary antibodies (926-32213 and 926-68022, LI-COR Biosciences) diluted at 1:10,000. Imaging and protein levels were quantified using the Odyssey system (LI-COR Biosciences).

### LysoTracker staining

Fibroblasts were isolated from ear tissue as previously described (Khan and Gasser, 2016). Cell cultures were established from each animal (*Npc1^+/+^*, *n*=2; *Npc1^em/em^*, *n*=2). LysoTracker staining and FACS analysis was performed on samples obtained on different culture days, as previously described (Rodriguez-Gil et al., 2013), for a total of six technical replicates for each animal. Fold change in LysoTracker staining was calculated as the ratio of geometric means of stained/unstained samples.

### GSL analysis

GSLs were analyzed essentially as previously described (Neville et al., 2004). Lipids from tissue homogenates were extracted with chloroform:methanol (1:2, v/v) overnight at 4°C. The GSLs were further purified using solid-phase C18 columns (Telos, Kinesis). After elution, the GSL fractions were dried under a stream of nitrogen and treated with recombinant endoglycoceramidase (rEGCase was kindly provided by Orphazyme) to obtain oligosaccharides from GSLs. The liberated glycans were then fluorescently labeled with anthranillic acid (2AA). Excess 2AA-label was removed using DPA-6S SPE columns (Supelco). Purified 2AA-labeled oligosaccharides were separated and quantified by normal-phase high-performance liquid chromatography (NP-HPLC) as previously described (Neville et al., 2004). The NP-HPLC system consisted of a Waters Alliance 2695 separations module and an in-line Waters 2475 multi λ-fluorescence detector set at Ex λ360 nm and Em λ425 nm. The solid phase used was a 4.6×250 mm TSK gel-Amide 80 column (Anachem). A 2AA-labeled glucose homopolymer ladder (Ludger) was included to determine the glucose unit values (GUs) for the HPLC peaks. Individual GSL species were identified by their GU values and quantified by comparison of integrated peak areas with a known amount of 2AA-labeled BioQuant chitotriose standard (Ludger). Results for tissue homogenates were normalized to protein content, determined by the bicinchoninic acid (BCA) assay.

### Tissue histology and immunohistochemistry

Tissues stored in 70% ethanol were processed for histology. Briefly, samples were dehydrated through graded alcohols, cleared in xylene and infiltrated with paraffin. After processing, all tissues were embedded in paraffin. The paraffin blocks were cut on a microtome at 5 μm. For H&E staining, the unstained slides were deparaffinized through xylene and graded alcohols to water, stained in hematoxylin, then rinsed again in water. The slides were then placed in 95% ethanol before staining with eosin, dehydrating through graded alcohols to xylene, then mounting with Permount (Thermo Fisher Scientific).

For specialized immunohistochemical staining of Purkinje cells in the cerebellum, anti-calbindin D antibody (C9848, Millipore Sigma) was used at a 1:1000 dilution, and mouse anti-Ig antibody (#BA-1000, Vector Laboratories; 1:500) was used as a secondary antibody with biotin. For immunohistochemical staining of macrophages in the liver and spleen, anti-CD68 (rabbit polyclonal, ab125212, Abcam) was used at a 1:120 dilution, and mouse Ig was used as a secondary antibody with biotin.

### Microscopy and CD68 quantification

Immunohistochemical images were collected using a Zeiss AxioScan.Z1 slide scanning microscope system (Carl Zeiss) with a Plan-Apochromat 20×/0.8 objective lens. All images were acquired using a Hitachi HV-F202 SCL camera with an average tile count of 200 tiles per liver section. The Zeiss ZEN blue 2.3 software package was used for collection and stitching. Immunohistochemical images were post-processed using MediaCybernetics’ Image-Pro Premiere 3D 9.3.3 software package. Every image was processed using multiple regions of interest (ROIs) each with an area of 75,649 µm^2^. Smart Segmentation was used to separate the presence of CD68 (ab125212, Abcam) by 3,3′-Diaminobenzidine (DAB) (D5905, Millipore Sigma) brown staining from blue-stained nuclei within the ROI. Finally, the Count/Size function was designed to extract the total counts, areas and total areas. Blinded counts of at least nine independent fields in nine ROIs per animal were used as the average percentage CD68^+^ area.

### Weight loss measurements and behavioral analysis

Weights were taken longitudinally beginning soon after weaning, and age of weight loss onset was identified as the first day of weight reduction. These numbers were then averaged for females and males. These were not recorded daily nor at a specific age, resulting in different longitudinal data points among animals.

For behavioral analysis, mice were scored weekly starting at 4 weeks of age based on the following six categories, which were previously published in two independent studies (Alam et al., 2016; Guyenet et al., 2010): grooming, gait, kyphosis, ledge test, hindlimb clasp and tremor. Each category had a range score from zero, meaning no phenotype, to three, meaning the most severe phenotype. A composite score for each animal was obtained by adding the scores for each of the six behavioral categories, resulting in a composite score range of 0 to 18.

### Lifespan measurement

Each litter was born to a mother that was previously separated from a male once a vaginal plug was identified or the female was noticeably pregnant. Animals were weighed weekly starting at weaning (P28). The maximum weight was recorded, and lifespan was defined by NHGRI Animal Care and Use Committee-approved end-point criteria of 30% weight loss from the maximum weight. Animals were euthanized, and tails were collected for DNA extraction.

### Generation of congenic BALB/cJ *Npc1^em^* mice

*Npc1^em/+^* heterozygote mice on a C57BL/6J background were backcrossed to BALB/cJ (Stock number: 000664, Jackson Laboratories) for six generations using marker-assisted selection/speed congenics techniques, briefly described as follows. Male *Npc1^em/+^* heterozygotes were selected for genotyping starting at the N2 backcross generation, and their genomes were assessed at the DartMouse™ Speed Congenic Core Facility (Geisel School of Medicine, Dartmouth College, New Hampshire, USA). Raw SNP data were analyzed using DartMouse's SNaP-Map™ and Map-Synth™ software, which determined the genetic background for each mouse at ∼3000 informative SNPs (from a custom panel selected by DartMouse) throughout the genome. Based on the genome scan results for the selected SNPs, the three *Npc1^em/+^* males with the highest percentage of BALB/cJ SNPs were selected as breeders for the next generation. The same steps were repeated at each subsequent generation. The selected males at the N3 generation exhibited 74.2-83.3% homozygosity for BALB/cJ SNPs, and the selected males at the N4 generation exhibited 92% homozygosity for BALB/cJ SNPs. For the N5 and subsequent generations, ≥92% of the SNPs would be homozygous for BALB/cJ, so males were bred for these generations without the need for genomic assessment. Mice were analyzed at the N4 generation [CB6J(B6JC)N4×CB6J(B6JC)N4; BALB/cJ N4] and the N6 generation [CB6J(B6JC)N6×C(B6JC)N6; BALB/cJ N6].

### Generation of *Npc1^em^* backcross mice and QTL linkage analysis

Backcross mice were generated by crossing a heterozygous *Npc1^em/+^* female (C57BL/6J background) to an *Npc1*^+/+^ BALB/cJ male, generating hybrid F1 mice heterozygous for variants from both strains. Heterozygous F1 *Npc1^em/+^* males were selected as breeders and backcrossed with heterozygous *Npc1^em/+^* females (C57BL/6J background), to generate the N2 backcross generation. Lifespan was measured for 202 N2 backcross homozygous *Npc1^em/em^* mutants, and DNA was collected from all 202 mice and subsequently used for QTL analysis. Samples were genotyped using GigaMUGA (Neogen) (Morgan et al., 2015). Non-informative variants were excluded based on control samples (Parental F1 and C57BL/6J only animals) as well as unreliable markers that failed in control samples or the majority of N2 samples. The full set of genotyping SNPs was filtered to obtain 28,873 genome-wide SNP markers that were informative between C57BL/6J and BALB/cJ. A mouse map converter (http://churchill-lab.jax.org/mousemapconverter) was used to convert the position of each SNP marker from base pair (GRCm38/mm10) to cM (Sex Averaged cM-Cox) (Cox et al., 2009; Liu et al., 2014). QTL linkage analysis was performed using R/qtl2 (Broman et al., 2019). One QTL genome scan was performed, selecting lifespan as the phenotype with a normal distribution. A permutation test (10,000 permutations) was carried out to obtain a 5% significance threshold (LOD=3.09).

### Analysis of candidate variants in QTL genomic regions

Genomic regions of interest for each of the two significant QTL peaks were defined using a 1.5-LOD support interval flanking the SNP with the maximum LOD score, as previously recommended (Manichaikul et al., 2006). The Mouse Genomes Project (Wellcome Sanger Institute, Hinxton, UK) was used to identify all known SNPs, indels and structural variants (SVs) within each genomic region that are informative between C57BL/6J and BALB/cJ. The following filters were selected to retain SNPs most likely to impact protein function: missense variant, splice acceptor variant, splice donor variant, splice region variant, stop gained and stop loss.

### Statistical analysis

Statistical analyses were carried out using Prism software (GraphPad). For Figs 1B, 2A, 4B and Fig. S2, unpaired Student's *t*-tests were performed. For Fig. 2B, a nested Student's *t*-test was performed. For Fig. 3B, two-way ANOVA with repeated measures and Bonferroni's correction was performed. For Fig. 5A, an ordinary one-way ANOVA was performed with multiple comparisons, using C57BL/6J as the control group. All survival analyses were carried out using the log-rank test (Mantel-Cox). In all figures, *P≤0.05, **P≤0.01, ***P≤0.001 and ****P≤0.0001.

## Supplementary Material

Supplementary information
