# Supplementary material for: Genetic background modifies phenotypic severity and longevity in a mouse model of Niemann-Pick disease type C1
Source: Dis Model Mech. 2020 Mar 13;13(3):dmm042614. doi: 10.1242/dmm.042614 (PMC7075069; doi:10.1242/dmm.042614)
Supplement: Supplementary information [file dmm-13-042614-s1.pdf]

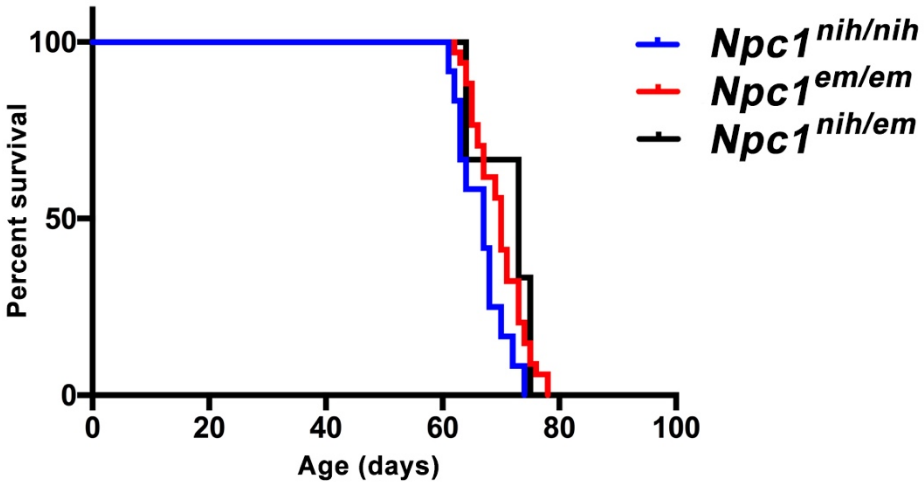

Figure S1. **Survival analysis of *Npc1$^{em/em}$* mice shows non-complementation between *Npc1^{em}* and *Npc1^{nih}* alleles.** A complementation test between *Npc1^{nih}* and *Npc1^{em1Pav}* (abbreviated *Npc1^{em}*) was done by generating *Npc1$^{nih/em}$* compound heterozygous mice (black line). No differences in lifespan were observed between *Npc1$^{nih/nih}$* vs. *Npc1$^{nih/em}$* (p=0.12), or *Npc1$^{em/em}$* vs. *Npc1$^{nih/em}$* (p=0.71).

Disease Models & Mechanisms • Supplementary information

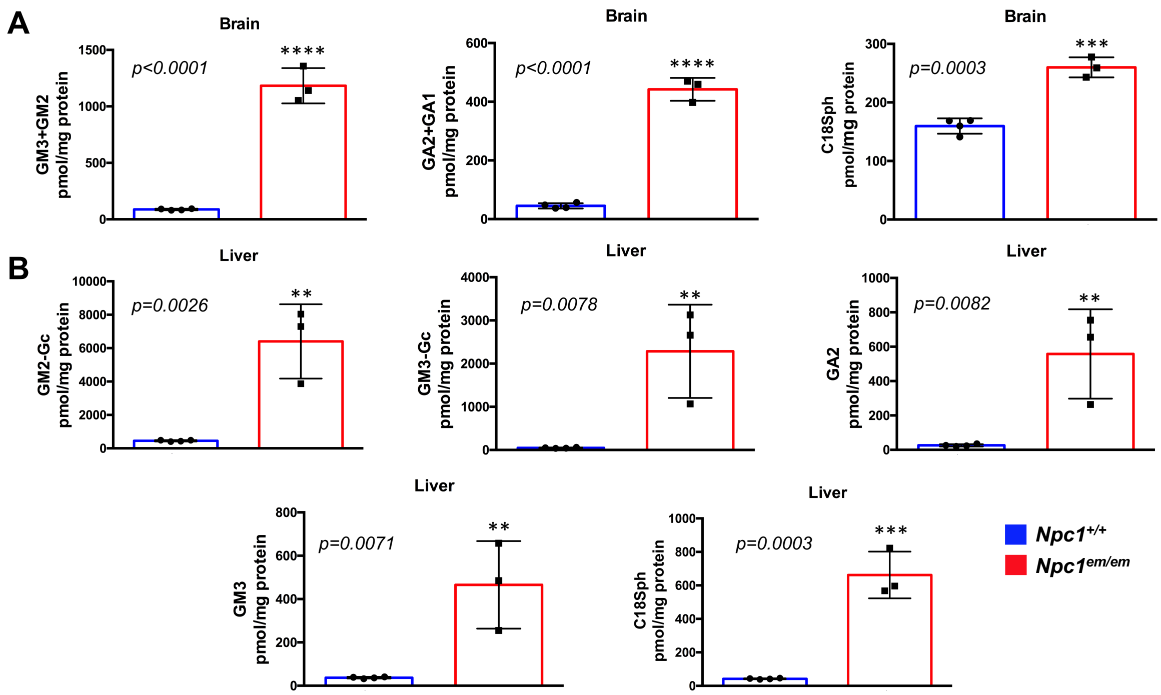

Figure S2. Glycosphingolipid (GSL) and sphingosine analysis shows abnormal lipid profiles in *Npc1*$^{em/em}$ mutants. Brain A) and liver B) were collected from controls (*Npc1*$^{+/+}$, n = 4) and *Npc1*$^{em1Pav/em1Pav}$ mutants (abbreviated *Npc1*$^{em/em}$, n=3), between P63 and P68. All animals were on a C57BL/6J genetic background. *Npc1*$^{em/em}$ mutants showed significantly elevated levels of individual GSLs and C18-sphingosine as compared to controls (*Npc1*$^{+/+}$). Brain and liver express different patterns of GSLs, thus the highest expressed GSLs were analyzed for each tissue. GM3, GM2, GM2-Gc and GM3-Gc are a-series gangliosides. GA2 and GA1 are o-series gangliosides. C18Sph = C18-sphingosine.

Disease Models & Mechanisms • Supplementary information

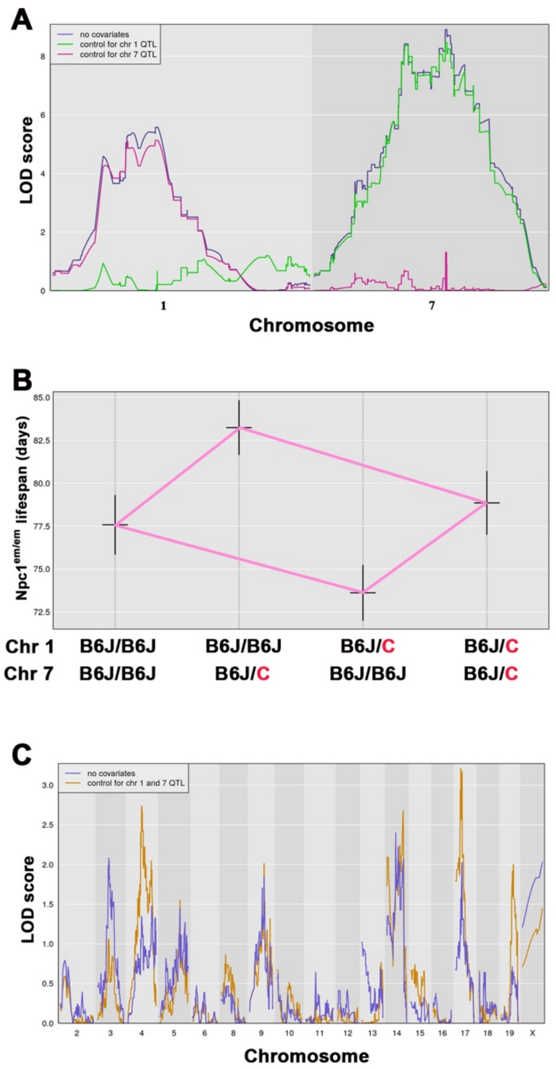

Figure S3. Analysis of the effects of the chromosome 1 and 7 QTLs. A) When controlling for chromosome 1 (green line), no additional QTLs are found within the same chromosome. The same results are shown for chromosome 7 (pink line). B) A plot of the estimated effects in each of the four genotype groups from the N2 backcross, showing the averages ± 2 SEs, indicates additivity between the two QTLs. The effect of the chromosome 1 QTL is similar, regardless of the genotype at the QTL on chromosome 7 (B6J/B6J or B6J/C) and *vice versa*, as indicated by the fact that the shape formed by the pink line segments is very nearly a parallelogram. B6J = C57BL/6J, C (in red) = BALB/cJ. C) When controlling for the genotypes of both chromosomes 1 and 7 (orange) there is significant linkage (LOD>3) detected on chromosome 17.

Table S1. Candidate variants from QTL regions on chromosomes 1 and 7 (in separate excel file).

Click here to Download Table S1

Table S2. Potential off-target sites from CRISPR/Cas9 sequence showed mismatch sequences with a low score.

Sequence*	Pam	Score	Gene	Locus
GCTAATAGCCAGTAACATCA	CGG	100	*Npc1* (ENSMUSG00000024413)	chr18:-12195061
GTTAATAGAAAGTAACATCA	AAG	1.6		chr13:-89173267
GCTTCTAGTCAGTAACATCA	AAG	1.6		chr2:+66840379
CCAAATATCTAGTAACATCA	CAG	1.5		chr9:-112090883
GCACATGACCAGTAACATCA	GGG	0.9		chr2:-57858396
TGTTATAGCCTGTAACATCA	CAG	0.9		chr13:-62601476
TGTTATAGCCTGTAACATCA	CAG	0.9		chr13:-62515053
CGTTATAGCCTGTAACATCA	CAG	0.9		chr13:-62366426
TGTTATAGCCTGTAACATCA	CAG	0.9		chr13:+62176500
TGTTATAGCCAGTAACATCA	CAG	0.9		chr13:+62053639

*Base-pairs in red denote mismatch with the original target sequence (top).

Disease Models & Mechanisms • Supplementary information
